# Correction: Comprehensive Reference Ranges for Hematology and Clinical Chemistry Laboratory Parameters Derived from Normal Nigerian Adults

**DOI:** 10.1371/journal.pone.0100601

**Published:** 2014-06-12

**Authors:** The PLOS ONE Staff

## Notice of Republication

This article was republished on May 28, 2014, to correct errors in several author names and affiliations. Please download this article again to view the correct version. The originally published, uncorrected article and the republished, corrected article are provided here for reference.

## Supporting Information

File S1 **Originally published, uncorrected article.**
(PDF)

File S2 **Republished corrected article.**
(PDF)
