# Supplementary material for: Correction: Comprehensive Reference Ranges for Hematology and Clinical Chemistry Laboratory Parameters Derived from Normal Nigerian Adults
Source: PLoS One. 2014 Jun 12;9(6):e100601. doi: 10.1371/journal.pone.0100601 (PMC4055763; doi:10.1371/journal.pone.0100601)
Supplement: File S1 — Originally published, uncorrected article. (PDF) [file pone.0100601.s001.pdf]

OPEN ACCESS Freely available online

Comprehensive Reference Ranges for Hematology and Clinical Chemistry Laboratory Parameters Derived from Normal Nigerian Adults

Timzing Miri-Dashe[1,4]*, Sophia Osawe[1,4], Monday Tokdung Nenbammun Daniel[2,1], Rahila Pam Choji[1], Ille Mamman[1,5], Kurt Deme[3], Dapus Damulak[3], Alash'le Abimiku[1,4,5]

1 Clinical Laboratory Services and Laboratory Research, Plateau State Human Virology Research Centre (PLASVIREC), Institute of Human Virology, Jos, Plateau State, Nigeria, **2** Medical Laboratory Science Council of Nigeria, Abuja, Nigeria, **3** National Blood Transfusion Service Jos, Plateau State Specialist Hospital # 13, Plateau State, Nigeria, **4** Clinical Laboratory Services, Institute of Human Virology, Abuja, Nigeria, **5** Division of Epidemiology and Prevention, Institute of Human Virology, University of Maryland, School of Medicine, Baltimore, Maryland, United States of America

Abstract

Background: Interpretation of laboratory test results with appropriate diagnostic accuracy requires reference or cutoff values. This study is a comprehensive determination of reference values for hematology and clinical chemistry in apparently healthy voluntary non-remunerated blood donors and pregnant women.

Methods and findings: Consented clients were clinically screened and counseled before testing for HIV, Hepatitis B, Hepatitis C and Syphilis. Standard national blood donors' questionnaire was administered to consented blood donors. Blood from qualified volunteers was used for measurement of complete hematology and chemistry parameters. Blood samples were analyzed from a total of 383 participants, 124 (32.4%) males, 125 (32.6%) non-pregnant females and 134 pregnant females (35.2%) with a mean age of 31 years. Our results showed that the red blood cells count (RBC), Hemoglobin (HB) and Hematocrit (HCT) had significant gender difference ($p = 0.000$) but not for total white blood count ($p > 0.05$) which was only significantly higher in pregnant verses non-pregnant women ($p = 0.000$). Hemoglobin and Hematocrit values were lower in pregnancy ($P = 0.000$). Platelets were significantly higher in females than men ($p = 0.001$) but lower in pregnant women ($p = 0.001$) with marked difference in gestational period. For clinical chemistry parameters, there was no significant difference for sodium, potassium and chloride ($p > 0.05$) but gender difference exists for Bicarbonate (HCO3), Urea nitrogen, Creatinine as well as the lipids ($p < 0.05$). Total bilirubin was significantly higher in males than females ($p = 0.000$). Significant differences exist for all chemistry parameters between pregnant and non-pregnant women in this study ($p < 0.05$), except Amylase and total cholesterol ($p > 0.05$).

Conclusions: Hematological and Clinical Chemistry reference ranges established in this study showed significant gender differences. Pregnant women also differed from non-pregnant females and during pregnancy. This is the first of such comprehensive study to establish reference values among adult Nigerians and difference observed underscore the need to establish reference values for different populations.

Citation: Miri-Dashe T, Osawe S, Daniel MTN, Choji RP, Mamman I, et al. (2014) Comprehensive Reference Ranges for Hematology and Clinical Chemistry Laboratory Parameters Derived from Normal Nigerian Adults. PLoS ONE 9(5): e93919. doi:10.1371/journal.pone.0093919

Editor: Kevan L. Hartshorn, Boston University School of Medicine, United States of America

Received October 3, 2013; **Accepted** March 11, 2014; **Published** May 15, 2014

Copyright: © 2014 Miri-Dashe et al. This is an open-access article distributed under the terms of the Creative Commons Attribution License, which permits unrestricted use, distribution, and reproduction in any medium, provided the original author and source are credited.

Funding: PLASVIREC funded this study through funds generated independently. Support was also received from the Institute of Human Virology Nigeria through supply of reagents for the testing. The funders had no role in study design, data collection and analysis, decision to publish, or preparation of the manuscript.

Competing Interests: The authors have declared that no competing interests exist.

* E-mail: tdashe@ihvnigeria.org

Introduction

Clinical laboratory tests are useful for diagnosing health disorders, drug toxicity or side effects, disease staging and monitoring of response to treatment. Interpretation of laboratory test results with appropriate diagnostic accuracy requires reference or cutoff values. In addition, the ability to enroll into clinical studies and the interpretation of results from those studies all hinge on using the appropriate reference values. Although several studies have been conducted in Nigeria to determine reference values for the immunologic indices such as CD4 T Lymphocytes [1,2] not many studies have established comprehensive hematology and chemistry reference values for adult Nigerians and the values are derived largely from other developed countries as reported in a number of studies that compared African volunteers with those from developed countries [3–7]. Studies conducted so far in Nigeria reported significant differences in normal laboratory ranges compared with those of other African countries and industrialized world [1] which could be due to a number of reasons including differences in geographical locations, climate, dietary habits, and environmental factors or ethnic and tribal peculiarities. These suggest that the development of normal values specific for Nigerian population is critical for interpretation of laboratory test results and provision of quality services in the Health care delivery. The aim of this study was to determine

reference values for hematology and clinical chemistry in apparently normal adults in Nigeria.

Materials and Methods

Study Design, Setting and Population

A cross sectional study was conducted in Jos, north central Nigeria. Participants were recruited from voluntary non remunerated blood donors at the National Blood Transfusion Service, Jos and pregnant women attending ante natal care (ANC) at Plateau state specialist Hospital. Blood samples were obtained from a cross sectional group of volunteers during blood donation drive activities of the National Blood Transfusion Service (NBTS) across Plateau state and neighboring states, including Nasarawa and Benue states; North Central and Gombe, and Bauchi states; North East Nigeria. Similarly, apparently healthy pregnant women were recruited from the Plateau State Specialist Hospital (PSSH) antenatal clinic (ANC) in Jos into the study. A total of 428 voluntary non-remunerated blood donors and ANC women between the ages of 18 and 65 years consented for the study.

Study Procedures

An expedited ethical clearance for this study was obtained from the Plateau State Specialist Hospital (PSSH) ethics committee as samples received for this study were aliquots that were coded. The NBTS team received written informed consent from all potential volunteers using the standard national donor questionnaire, before blood draws. Blood donors were bled at NBTS and aliquots of these samples were sent for screening and analysis for this study at PLASVIREC. Samples from ANC women were also collected after consenting by another study at PLASVIREC who were enrolling antenatal women into a prospective cohort study. Samples from these women were also screened and analyzed for this study. All sample aliquots received at PLASVIREC were coded with no linking identifiers to the blood donors or antenatal women.

Additional questionnaires were used by the NBTS team to capture demographic data, medical status, medical history and social habits of each blood donor. Volunteers who screened positive for any of the transmissible infections, or, donated or received blood transfusion in the previous month and those who failed to give consent were excluded from the study. Results of HIV test were given to all participants after post-test counseling by trained HIV counselors at NBTS and PSSH.

Blood Collection and Serology Tests

Blood samples were collected using ethylene diamine - tetra - acetic acid (EDTA) vacutainer tubes for CD4 and complete blood count. Another blood sample in plain vacutainer tubes was collected for transfusion transmissible infections screening (HIV rapid test, HBsAg, HCV and Syphilis) and chemistry assays. Blood samples were transported in cold chain boxes to the Plateau State Human Virology Research Centre (PLASVIREC) for CD4 counts and hematology assays, done within six hours of sample collection. The serum was separated immediately and assayed within 8 hours of collection or stored at −20°C for later chemistry assay. An aliquot of the serum from each volunteer was also transported to National Blood Transfusion service (NBTS) for testing for HIV, Hepatitis B (HBsAg), Hepatitis C and syphilis. HIV rapid testing was also done at PLASVIREC with the aliquot used for the chemistry testing.

Table 1. CD4 absolute Count for normal adults and pregnant women in Nigeria.

Source	Sex	N	Mean	SD	95% CI	Median	Range	2.5th–95th percentile
Blood donors	Combined	380	723.53	205.13	702.8–744.2	691.5	314–1347	572.0–1127.3
	Male	122	711.36	207.88	674.1–748.6	672.5	314–1347	554.5–1087
	Female	127	778.27	213.25	740.8–815.7	754.0	358–1328	627.0–1274.0
Pregnant women	Female	131	681.79	183.19	650.1–713.5	657.0	332–1331	551.0–1017.6

P-value 0.001.
doi:10.1371/journal.pone.0093919.t001

Table 2. Hematological parameters for normal adult Nigerians.

Subject	Parameter	WBC ($X10^3$/UL)	RBC ($X10^6$/UL)	HB (g/dl)	HCT (%)	MCV (fl)	MCH (pg)	MCHC (g/dl)	PLT (10_3/ul)	LYM (%)	NEUT (%)	MONO (%)	BASO (%)	EOSIN (%)
**Male**	Mean	4.40	5.20	14.20	44.19	85.52	27.66	32.18	216.40	39.02	53.54	5.84	0.09	1.50
	Ref. range	4.3–4.6	5.1–5.3	14.0–14.4	43.5–45.0	84.3–86.6	27.2–28.1	31.9–32.4	206.8–226.8	37.4–40.2	52.6–552	5.3–6.4	0.02–0.12	1.1–1.9
	Median	4.40	5.20	14.30	44.80	85.60	27.90	32.40	213.00	40.00	53.00	6.00	0.00	1.00
	S. D.	0.86	0.72	1.31	3.99	6.31	2.58	1.33	54.51	7.60	7.34	2.98	0.34	1.94
**Female**	Mean	4.61	4.60	12.75	39.73	85.81	27.57	32.06	240.95	40.32	50.69	7.02	0.12	1.63
	Ref. range	4.4–4.8	4.5–5.3	12.4–13.1	38.8–40.5	84.8–86.5	27.1–28.9	31.8–32.3	229.3–251.2	39.0–42.1	49.1–52.3	6.5–7.5	0.06–0.18	1.32–1.91
	Median	4.50	4.56	12.80	40.00	86.10	27.40	32.10	236.00	41.00	51.00	7.00	0.00	2.00
	S. D.	1.05	0.63	1.69	4.68	4.90	2.077	1.17	59.63	8.80	9.30	2.82	0.33	1.63
	**P-value**	0.083	0.000	0.000	0.000	0.686	0.775	0.426	0.001	0.215	0.008	0.002	0.446	0.583

doi:10.1371/journal.pone.0093919.t002

Table 3. Hematological parameters for pregnant and non- pregnant women in Nigeria.

Subject	Parameter	WBC ($X10^3$/UL)	RBC ($X10^6$/UL)	HB (g/dl)	HCT (%)	MCV (fl)	MCH (pg)	MCHC (g/dl)	PLT (10_3/ul)	LYM (%)	NEUT (%)	MONO (%)	BASO (%)	EOSIN (%)
**Pregnant female**	Mean	6.62	4.27	11.80	36.00	85.02	27.66	32.75	215.44	27.27	63.61	7.27	0.08	1.84
	Ref. range	6.4–6.9	4.2–4.4	11.6–12.0	35.6–36.5	83.9–86.1	27.0–28.3	32.5–33.0	205.6–225.6	26.2–28.2	62.7–64.8	6.9–7.6	0.03–0.12	1.5–2.1
	Median	6.40	4.22	11.90	36.00	84.90	27.90	32.80	207.00	26.00	64.00	7.00	0.00	2.00
	S. D.	1.50	0.45	0.97	2.66	6.24	3.53	1.29	58.432	5.89	6.25	1.90	0.28	1.75
**Non- pregnant female**	Mean	4.61	4.60	12.75	39.73	85.81	27.57	32.06	240.95	40.32	50.69	7.02	0.12	1.63
	Ref. range	4.43–4.82	4.5–5.3	12.4–13.1	38.8–40.5	84.8–86.5	27.1–28.9	31.8–32.3	229.3–251.2	39.0–42.1	49.1–52.3	6.5–7.5	0.06–0.18	1.32–1.91
	Median	4.5	4.56	12.80	40.00	86.10	27.40	32.10	236.00	41.00	51.00	7.00	0.00	2.00
	S. D.	1.05	0.63	1.69	4.68	4.90	2.077	1.17	59.63	8.80	9.30	2.82	0.33	1.63
	**P-value**	0.000	0.00	0.000	0.000	0.262	0.520	0.000	0.001	0.000	0.000	0.411	0.301	0.314

doi:10.1371/journal.pone.0093919.t003

Table 4. Hematological parameters at different trimester for pregnant women in Nigeria.

Trimester	Parameter	WBC ($X10^3$/UL)	RBC ($X10^6$/UL)	HB (g/dl)	HCT (%)	MCV (fl)	MCH (pg)	MCHC (g/dl)	PLT (10_3/ul)	LYM (%)	NEUT (%)	MONO (%)	BASO (%)	EOSIN (%)
First	Mean	5.89	4.72	11.81	37.69	80.91	25.05	31.30	249.77	29.92	59.85	8.15	.15	1.92
	Ref. range	5.2–6.6	4.5–5.0	11.1–12.5	35.9–39.4	77.4–84.5	23.4–26.7	30.5–32.1	221.8–277.8	26.8–33.0	56.9–63.1	7.1–9.3	−0.07–0.38	0.8–3.0
	Median	5.80	4.67	11.40	37.00	81.40	25.50	31.70	250.00	29.00	60.00	9.00	0.00	2.00
	S. D.	1.13	.43	1.10	2.88	5.86	2.68	1.33	46.37	5.11	5.40	1.82	.38	1.80
Second	Mean	6.92	4.14	11.54	35.47	86.06	28.01	32.52	234.49	27.41	64.03	7.16	.11	1.30
	Ref. range	6.4–7.4	4.0–4.3	11.3–11.8	34.8–36.2	84.1–88.0	27.2–28.8	32.2–32.9	212.9–256.1	25.8–29.0	62.3–65.8	6.6–7.8	0.00–0.2	0.8–1.8
	Median	7.10	4.19	11.70	35.60	87.10	28.00	32.60	220.00	28.00	64.00	7.00	0.00	1.00
	S. D.	1.53	0.35	.80	2.14	5.94	2.40	1.00	64.82	4.75	5.23	1.85	.32	1.49
Third	Mean	6.61	4.26	11.91	36.00	85.20	27.90	33.08	201.58	26.80	64.01	7.18	.06	2.07
	Ref. range	6.3–6.9	4.2–4.4	11.7–12.1	35.4–36.6	83.8–86.6	27.0–28.8	32.8–33.4	190.1–212.9	25.3–28.1	62.8–65.6	6.7–7.6	0.00–0.10	1.6–2.5
	Median	6.55	4.20	12.00	36.30	85.10	28.20	33.10	198.00	26.00	65.00	7.00	0.00	2.00
	S. D.	1.50	.459	1.00	2.75	6.25	3.91	1.23	52.84	6.38	6.64	1.92	.24	1.81
	P values	0.100	0.000	0.145	0.033	0.033	0.018	0.000	0.100	0.203	0.072	0.212	0.426	0.079

doi:10.1371/journal.pone.0093919.t004

Quality Control

PLASVIREC Laboratory is a 5 star laboratory according to the WHO/AFRO step wise accreditation ranking which is based on ISO 15189. In addition, the lab routinely participates in a number of external proficiency testing panels including TISTLE QA South Africa PT, and Digital PT Canada in addition to the quarterly internal proficiency testing. Quantitative QC was run daily or each time an assay was run and values were plotted on LJ charts and results were verified using Wetsgard rules. In the event that the daily commercial controls or in house prepared controls failed, testing was suspended until evaluated.

Laboratory Assays

HIV, Syphilis, Hepatitis B and Hepatitis C Serology Testing

Serum blood samples were initially screened for HIV, Syphilis, Hepatitis B and Hepatitis C using ELISA techniques at the National Blood Transfusion Service (NBTS) Jos. For HIV screening, Bio-Rad gensreen ultra HIV Ag-Ab, a semi-automated 4th generation ELISA technique was used. Hepatitis B was screened using Bi-rad monolisa HBsAg ultra method; and Hepatitis C screening was done using DIA.PRO HCV Ab; a third generation enzyme immunoassay for the determination of anti-Hepatitis C Virus antibody in human serum and plasma. Syphilis test was done using SYPH Ab immunoassay method for the detection of antibodies to *Treponema, pallidum* in human serum. The NBTS program was also interested in testing the performance of rapid test kits against the conventional ELISA method so rapid test techniques were also used for HIV, Syphilis, Hepatitis B and Hepatitis C Serology to compare the sensitivity of rapid techniques with ELISA. HIV rapid test were carried out using the Nigerian HIV parallel testing algorithm made of a combination of three test kits (Determine, Unigold and Stat-pak) approved by the National Agency for Food Drugs Administration Control (NAFDAC).

Hematology Assay

A complete blood count (CBC) and differential was performed on the blood sample using Sysmex KX-21N, an automated 3-part differential hematology analyzer (Sysmex Corporation Kobe, Japan). The machine automatically dilutes whole-blood sample of 50 μl in the CBC/Differential mode, lyses and enumerates white blood cells (WBC), red blood cells (RBC), hemoglobin concentration (Hb), pack cells volume (PCV), platelets, lymphocytes, neutrophils and the red cells indices (MCV, MCH & MCHC). It however does not count for eosinophil and basophils counts. Therefore, manual differential count was done on well prepared thin blood films.

Clinical Chemistry Assay

Serum Samples were analyzed using the Vitros 350 fully automated Chemistry Analyzer (Ortho-clinical Diagnostic) for key analytes according to the manufacturer's Instructions and standard operating procedures (SOP). The V350 analyzer is high throughput Vitros equipment, which operates on the dry slide technology. The unique advantage of these slides is the elimination of the need for storage, mixing and disposing of liquid chemicals reagents while permitting reliable analyses small patient sample (5–11 μl) per test.

Statistical Methods

Reference ranges were calculated using nonparametric methods. The medians were calculated and reference values were determined at 2.5th and 95th percentiles. Mean, median and standard deviations were computed for each of the clinical

Table 5. Hematological Reference ranges comparison with other African countries.

	Parameter	Nigeria (This study)	Botswana [8]	Kenya [5]	Uganda [9]	U S [11]
**FEMALES**	WBC ($X10^3$/UL)	4.4–4.8	N/A	N/A	N/A	N/A
	RBC ($X10^6$/UL)	4.5–5.3	3.7–5.1	3.7–5.6	3.3–5.3	4.0–5.2
	HB (g/dl)	12.4–13.1	9–15	5.9–10.0	9.8–16.2	12.0–16.0
	HCT (%)	38.8–40.5	29–43	30–50	28.3–46.8	36–46
	MCV (fl)	84.8–86.5	65–95	66.0–95.7	74–94.5	N/A
	MCH (pg)	27.1–28.9	20–32	21.3–33.0	24.8–32.7	-
	MCHC (g/dl)	31.8–32.3	31–37	32.2–35.3	33–35.5	-
	PLT (10_3/ul)	229.3–251.2	N/A	N/A	N/A	N/A
	Lymphocyte (%)	39.0–42.1	N/A	N/A	N/A	N/A
	Neutrophils (%)	49.1–52.3	N/A	N/A	N/A	N/A
	Monocyte (%)	6.5–7.5	N/A	N/A	N/A	N/A
	Basophils (%)	0.06–0.18	N/A	N/A	N/A	N/A
	Eosinophils (%)	1.32–1.9	N/A	N/A	N/A	N/A
**MALES**	**WBC ($X10^3$/UL)**	4.3–4.6	3–10	2.8–8.2	2.8–8.2	4.5–11.0
	RBC ($X10^6$/UL)	5.1–5.3	4.4–6.0	4.4–6.3	3.8–6.1	4.5–5.9
	HB (g/dl)	14.0–14.4	13–17	8.3–11.3	11.6–17.1	13.5–16.0
	HCT (%)	43.5–45.0	38–49	40–50	33.8–49.5	41–53
	MCV (fl)	84.3–86.6	76–93	71.4–98.2	71–97	80–100
	MCH (pg)	27.2–28.1	24–33	23.3–33.8	23.0–33.8	-
	MCHC (g/dl)	31.9–32–4	31–37	32.2–35.2	32.4–35.3	-
	PLT (10_3/ul)	206.8–226.8	160–395	120–411	109–384	150–350
	Lymphocyte (%)	37.4–40.2	20–54	20–60	26.7–61.2	22–44
	Neutrophils (%)	52.6–55.2	33–69	40–60	22.2–59.3	40–70
	Monocyte (%)	5.3–6.4	4–13	3–11	4.7–12.7	4–11
	Basophils (%)	0.02–0.12	0–2	0–2	0.3–1.4	0–3
	Eosinophils (%)	1.1–1.9	0–9	1–20	1.0–25	0–8

doi:10.1371/journal.pone.0093919.t005

chemistry and hematological parameters of the study subjects. Parametric student's t-test was used to determine any statistically significant differences between males and non-pregnant females; as well as pregnant and non-pregnant females. The analysis of variance (ANOVA) was employed for any statistical significant difference in these parameters in relation to the trimesters. $P < 0.05$ was considered significant. All statistical analyses were done using SPSS version 17.0.

Results

Screening

Of the 428 participants consented for the study, 2 (0.46%) were HIV positive, 23 (5.4%) had Hepatitis B, 19 (4.4%) had Hepatitis C and 1 (0.23%) had Syphilis. These were referred for standard care. Also, about 16 (3.4%) had CD4 outside the established reference range for the country by other studies [1,2]. The data of subjects in these groups were excluded from analysis.

Missing data accounted for 16 (3.7%). Complete data from 383 participants were included in the analysis. The mean age of all participants at study entry was 31 years. Men were slightly older than women, with mean ages of 33 and 30 years respectively. Mean age for pregnant women was 28 years.

For the purpose of comparative analysis of sensitivity of rapid technique against the ELISAs, all serologic tests (HIV, HBsAg, HCV& Syphilis) were performed using both techniques.

Immunologic Indices Analysis

The mean absolute CD4 T cell count was 723.53±95th percentile reference intervals (702.8–744.2 cells/μl) for blood donors. The mean absolute CD4 T cell count for pregnant females was 681.0 at 95th percentile reference interval (650.1–713.5 cells/μl) similar to the mean of 771 obtained in our previous study and significantly lower than non-pregnant females and males 778.27(627–1274.0 cells/μl) and 711.36(554.0–1087.0 cells/μl) respectively (Table 1) also confirming our earlier study [1].

Hematological Indices

Tables 2, 3 and 4 show the mean, SD, median and 95% reference range (2.5th–95th percentiles) for hematological parameters according to gender and pregnancy status. The Red Blood Cells (RBC) count, Hemoglobin (HB) and Hematocrit (HCT) showed significant gender difference ($p = 0.000$). There was no statistically significant gender difference for total White Blood Cells (WBC) count ($p > 0.05$). Total WBC was however significantly higher in pregnant women than their non-pregnant counterparts ($p = 0.000$). The comparison of platelet count by gender showed higher values among the females ($p = 0.001$); with lower counts in pregnant women ($p = 0.001$). So also were the values for Hemoglobin and Hematocrit ($P = 0.000$). Gestational period in pregnancy revealed marked difference in platelet count ($p = 0.001$) with no significant difference in WBC. In addition,

Table 6. Clinical chemistry parameters for adult Nigerians.

Subject	Parameter	Na+(Mmol/L)	K+ (Mmol/L)	Cl− (Mmol/L)	HCO3 (Mmol/L)	Urea (Mmol/L)	Creatinine (μmol/L)	Glu (Mmol/L)
Male	Mean	133.80	4.78	99.90	24.55	2.95	85.82	4.93
	Reference range	132.0–149.4	4.0–7.5	98.0–108.4	22.0–32.0	2.2–4.8	76.3–111.1	3.7–7.9
	Median	136.0	4.6	100.0	24.0	2.7	86.0	4.9
	S. D.	11.26	1.23	4.54	3.97	0.95	14.45	1.57
Female	Mean	132.06	5.05	99.35	18.32	3.34	79.29	5.89
	Reference range	120.0–156.6	4.0–7.7	98.0–110.0	14.0–29.0	2.5–5.8	63.0–117.8	4.2–9.6
	Median	133.0	4.7	101.0	17.0	3.2	76.0	5.0
	S. D.	17.50	1.52	11.94	6.31	1.36	21.14	4.17
**P-value**		0.351	0.127	0.579	0.000	0.010	0.005	0.018

doi:10.1371/journal.pone.0093919.t006

Table 7. Clinical chemistry parameters for adult Nigerians (continue).

Subject	Parameter	AST (μ/L)	ALT (μ/L)	T/Bil (μmol/L)	Aml (μ/L)	TChol (Mmol/L)	Trig (Mmol/L)
**Male**	Mean	33.12	24.42	7.18	67.47	3.75	1.11
	Reference range	26.0–49.4	17.3–48.4	3.42–17.1	52.0–127.5	3.2–5.3	0.7–2.2
	Median	31.0	22.0	5.13	66.0	3.7	1.1
	S. D.	10.22	9.90	4.96	24.18	1.01	0.54
**Female**	Mean	32.59	24.13	2.34	73.96	3.85	0.99
	Reference range	22.0–58.4	19.0–38.0	0.3–10.6	55.0–122.4	3.1–5.6	0.6–2.1
	Median	31.0	23.0	1.9	73.0	3.8	0.8
	S. D.	11.78	7.38	3.41	25.99	1.11	0.56
P-value		0.710	0.790	0.000	0.042	0.426	0.073

doi:10.1371/journal.pone.0093919.t007

Table 8. Clinical Chemistry Parameters for pregnant and non-pregnant women in Nigeria.

Subject	Parameter	Na+ (Mmol/L)	K+ (Mmol/L)	Cl− (Mmol/L)	HCO3 (Mmol/L)	Urea (Mmol/L)	Creatinine (μmol/L)	Glu (Mmol/L)
Non-Pregnant Female	Mean	132.06	5.05	99.35	18.32	3.34	79.29	5.89
	Reference range	120.0–156.6	4.0–7.7	98.0–110.0	14.0–29.0	2.5–5.8	63.0–117.8	4.2–9.6
	Median	133.0	4.7	101.0	17.0	3.2	76.0	5.0
	S. D.	17.50	1.52	11.94	6.31	1.36	21.14	4.17
Pregnant Female	Mean	139.01	4.30	111.53	17.48	5.39	58.07	4.31
	Reference range	135.0–151.6	3.9–5.7	109.0–125.0	14.0–28.2	4.6–7.4	46.0–91.0	3.7–6.1
	Median	139.0	4.1	112.0	16.0	5.2	55.0	4.2
	S. D.	8.68	0.65	7.47	5.11	1.16	16.81	0.96
	**P-value**	0.000	0.000	0.000	0.239	0.000	0.000	0.000

doi:10.1371/journal.pone.0093919.t008

Table 9. Clinical Chemistry Parameters for pregnant and non-pregnant women in Nigeria.

Subject	Parameter	AST (μ/L)	ALT (μ/L)	T/Bil (μmol/L)	Aml (μ/L)	T/Chol (Mmol/L)	Trig (Mmol/L)
Non-Pregnant Female	Mean	32.59	24.13	2.34	73.96	3.85	0.99
	Reference range	22.0–58.4	19.0–38.0	0.3–10.6	55.0–122.4	3.1–5.6	0.6–2.1
	Median	31.0	23.0	1.9	73.0	3.8	0.8
	S. D.	11.78	7.38	3.41	25.99	1.11	0.56
Pregnant Female	Mean	18.61	32.01	8.41	71.16	3.97	1.28
	Reference range	13.3–32.9	28.0–46.0	6.0–16.9	56.0–111.2	2.0–6.3	1.0–2.5
	Median	19.0	33.0	7.6	71.0	4.5	1.3
	S. D.	7.30	9.60	3.75	21.10	1.82	0.58
	P-value	0.000	0.000	0.000	0.345	0.554	0.000

doi:10.1371/journal.pone.0093919.t009

Table 10. Clinical chemistry parameters for pregnant women at different trimesters in Nigeria.

Trimester	Parameter	Na+ (Mmol/L)	K+ (Mmol/L)	Cl− (Mmol/L)	HCO3 (Mmol/L)	Urea (Mmol/L)	Creatinine (µmol/L)	Glu (Mmol/L)	AST (µ/L)	ALT (µ/L)	T/Bil (µmol/L)	Aml (µ/L)	T/Chol (Mmol/L)	Trig (Mmol/L)
**First**	Mean	134.15	4.39	110.31	18.00	5.85	46.46	3.97	21.54	31.69	6.92	67.18	4.56	1.31
	Ref. range	134.0-	4.1-	105.0-	13.0-	5.6-	37.0-	3.4-	17.-	29.0-	6.0-	55.0-	4.0-	0.9-
	Median	138.0	4.4	111.0	20.0	5.8	51.0	3.8	22.0	31.0	7.0	72.0	4.5	1.3
	S. D.	12.73	0.88	6.22	5.92	1.18	11.01	1.05	8.66	9.11	1.98	13.36	0.98	0.51
**Second**	Mean	136.84	4.14	105.89	18.95	5.47	46.03	3.92	22.19	31.24	7.05	76.06	5.75	1.71
	Ref. range	135.0–142.3	3.9–5.3	10.3–115.3	14.0–29.5	4.3–8.5	41.0–59.3	3.4–6.4	18.8–32.3	28.8–46.8	5.0–15.8	58.8–116.8	5.3–7.1	1.3–3.1
	Median	137.0	4.0	108.0	17.5	5.0	45.0	3.8	21.5	31.0	6.0	77.0	5.7	1.6
	S. D.	2.45	0.45	7.51	5.61	1.32	6.34	0.86	4.50	5.90	3.28	19.86	0.73	0.63
**Third**	Mean	140.71	4.35	114.18	16.76	5.28	65.08	4.53	16.60	32.39	9.23	69.69	3.10	1.24
	Ref. range	135.0–152.0	3.9–5.7	111.0–127.0	14.0–25.9	4.7–7.0	54.0–93.9	4.0–6.1	10.8–32.7	27.8–46.7	7.6–17.8	52.8–109.6	1.7–6.1	0.9–2.3
	Median	142.5	4.2	114.0	15.0	5.2	65.5	4.5	15.05	34.0	7.6	69.5	2.4	1.1
	S. D.	9.27	0.68	6.17	4.65	1.08	16.78	0.92	7.39	10.94	3.93	22.23	1.65	0.50
**P value**		0.007	0.236	0.000	0.088	0.239	0.000	0.001	0.000	0.828	0.004	0.270	0.000	0.000

doi:10.1371/journal.pone.0093919.t010

platelet count decreased with pregnancy age (Table 4). Hematological parameters derived in this study were compared to reported ranges from other selected countries in Africa [5,8,9,10] and the US [11]. The Kenyan and Ugandan references derived as shown in **Table 5** have lower HB values and high HCT values. These values were explained by the authors to be due to diet and the effects of high altitudes [5]. The health status of the Ugandan population used in the study was not known and may account for the differences in HB, HCT and the broad ranges reported for MCV [9].

Chemistry Indices

Unlike the platelet counts, comparison by gender showed no significant difference for sodium, potassium and chloride ($p>0.05$). Gender difference however exists for Bicarbonate (HCO3), Urea nitrogen, creatinine as well as the lipids ($p<0.05$). Liver enzymes in this study show no significant variation except total bilirubin ($p = 0.000$), although values for female are slightly higher. Significant differences exist for all chemistry parameters between pregnant and non-pregnant women in this study ($p<0.05$) except Amylase, total cholesterol and bicarbonate ($p>0.05$) as illustrated in Tables 6, 7, 8, 9, 10 and 11.

Discussions

The results from this study has confirmed earlier findings by our group and others on the level of CD4 positive lymphocyte count in normal adult Nigerians compared to pregnant women [1] other hematological and clinical chemistry parameters, our study found a slightly higher reference range for CD4 counts in the blood donor population (572.0–1,127.3 cells/µl), comparable to the study of Urassa et al 2003 [12] (405–1,500 cells/µl). The slightly higher counts in blood donors may be due to the positive haemopoietic feedback stimulus following blood loss. Females subjects in our study had higher CD4 counts than males as earlier described by other investigators [2,8] with the non-pregnant women having higher CD4 count ranges than pregnant females.

A total of thirteen hematology parameters were tested in this study and showed significant differences among males and females as reported in other studies [4,5,8,13]. The red blood cell parameters, RBC, HB and HCT were significantly higher in male blood donors ($p = 0.000$). This finding is similar to reports from other studies conducted in Africa and also comparable to documentation from the United States of America [5]. On the contrary, platelet ranges were significantly higher in women as compared to men just as reported in other studies [5,8,14]. The reason for these differences may be due to the variations in hormone types and concentrations in the different sexes and the effect of erythropoietin release in response to regular menstruation cross-stimulating megakaryopoiesis. However, the platelet counts are lower when compared to the US derived values and other African studies [5,8,9,10,11]. The reason for these lower values is still unclear and may require additional studies but may be due to the diet, genetic factors or other environmental or genetic factors [3,15,16]. Unlike other African studies, we observed lower eosinophil and WBC values in our population [6,17,18]. When hematology ranges among pregnant and non-pregnant women were compared regardless of trimester, differences in reference ranges was observed in eight parameters; WBC, RBC, HB, HCT, MCHC, lymphocyte percentage, neutrophil percentage and platelets; all values were higher in pregnancy which may be due to hormonal changes, fluid retention or medication during pregnancy. Interestingly we reported significant difference in hematology parameters of RBC, MCHC and platelet ranges

Table 11. Chemistry Reference ranges comparison with other countries.

Parameter	Nigeria (This study)	Kenya (All)	Tanzania (All)	U.S (All)
Na+(Mmol/L) Males	133.8(132.0–149.4)	146.5(141.8–152.1)	(134–142)	(136–145)
Na+(Mmol/L) Females	132.1(120–156.2)	146.5(140.3–155.3)		
K+(Mmol/L) Males	4.6(4.0–7.5)	4.6(3.9–5.8)	(3.8–5.5)	(3.5–5.0)
K+(Mmol/L) Females	5.1(4–7.7)	4.5(3.8–5.8)		
Cl− (Mmol/L) Male	99.9(98.0–108.4)	105.0(100.4–110.8)	(98–107)	(98–106)
Cl− (Mmol/L) Females	99.4(98–110)	106.9(101–113.4)		
HCO3(Mmol/L) Males	24.4(22.0–32.0)	23.5(18.9–29.0)	(19–30)	(21–30)
HCO3(Mmol/L) Females	18.3(14–29)	21.6(16.9–26.9)		
Urea (Mmol/L) Males	3.0(2.2–4.8)	2.8(1.5–4.6)	(1.5–5.0)	(3.6–7.1)
Urea (Mmol/L) Females	3.3(2.5–5.8)	2.5(1.4–4.6)		
Creat (umol/L) Males	85.8(76.3–111.1)	77(62.0–106.0)	(0–90)	(0–133)
Creat (umol/L) Females	79.3(63–117.8)	66(51–91)		
Glu (Mmol/L) Males	4.9(3.7–7.9)	4.1(3.0–5.6)	(2.9–5.2)	(4.2–6.4)
Glu (Mmol/L) Females	5.9(4.4–9.6)	4(3.2–5.7)		
AST (U/L) Males	33.1(26.0–49.4)	23.9(14.9–45.3)	(0–48)	(0–35)
AST (U/L) Females	33(22–58.4)	19.1(13.1–38.1)		
ALT (U/L) Males	24.4(17.3–48.4)	22.3(10.8–53.9)	(0–48)	(0–35)
ALT (U/L) Females	24.1(19–38)	16.8(8.6–47)		
T/Bil (μmol/L) Males	6.8(3.4–17.1)	12.2(5.6–42.9)	(5.2–41)	(5.1–17.0)
T/Bil (μmol/L) Females	2.3(0.3–10.6)	9.6(4.4–26.8)		
Amyl (U/L) Males	68(52.0–127.5)	85(40–171.3)	(43–164)	(60–180)
Amyl (U/L) Females	74(55–122.4)	78.6(36–147.8)		
T/Chol (Mmol/L) Males	4.8(3.2–5.3)	3.8(2.5–5.5)	(0–5.5)	(0–6.2)
T/Chol (Mmol/L) Females	4(3.1–5.6)	3.9(2.6–5.9)		
Trig (Mmol/L) Males	1.1(0.7–2.2)	0.9(0.4–2.7)	(0–2.9)	(0–1.8)
Trig (Mmol/L) Females	1.0(0.6–2.1)	0.8(0.4–2.5)		

doi:10.1371/journal.pone.0093919.t011

among pregnant women based on their trimester. In addition when we compared hematology indices with those from the USA, our ranges are slightly higher. This underscores the need to establish reference levels of local populations and additional studies to confirm these interesting findings.

The clinical chemistry reference range data for electrolytes in this study is similar to one derived from Kenya [5], Ghana [14,19], Tanzania [4,20] and the USA [11]. Glucose and urea levels in Nigerians were higher than those found among Kenyans and Tanzanians but similar to those reported in subjects from the USA (Table 11). On the other hand, enzyme levels in the Nigerian population were higher than those recorded in the USA [11] but similar to those documented in other African countries [4,5], except amylase levels, which was significantly higher in the USA subjects [11]. Differences in values exist by gender as well as by country. Glucose and liver enzymes were significantly higher in Nigerian men than women while it was the opposite for amylase and urea levels. Total bilirubin values were significantly higher in males than females ($p = 0.000$), this agrees with findings from other African countries [5,19]. Our study also observed that Total bilirubin increases with gestational age. Significant differences exist for all electrolytes in pregnant and non-pregnant females. Urea level was elevated in pregnancy as seen in (Table 8).

As mentioned above, differences also exist across countries e.g. AST and ALT values from Nigerians were higher than those reported for Kenyans [5], Tanzanians [4], and USA [11]. Creatinine, urea and amylase reported in the USA were higher than those reported in Africans. The values from this study showed remarkable difference in hemoglobin and glucose reference range from those of African countries but similar to the USA.

In conclusion, laboratory reference ranges generated in this study are one of the most comprehensive hematology and clinical chemistry data sets produced in Nigeria. The significant differences between males and females, during pregnancy, and across countries in hematological and clinical chemistry reference ranges illustrated by this study underscore the need for such comprehensive establishment of reference values for different populations. The differences observed among pregnant women at the different trimesters was unexpected but underscores the role that hormonal changes could play in establishing the normal ranges for these parameters between genders and also during pregnancy. These findings are extremely valuable as reference values in the Nigerian populations for interpretation of laboratory results for patient management and clinical trials.

Acknowledgments

The authors thank all the study participants, staff of the National Blood Transfusion Service (NBTS) North Central Zonal Centre, Jos, and the research staff at Plateau State Human Virology Research Center (PLASVIREC). We will like to acknowledge Mr. Jalo Philip for his help in the data analysis.

Author Contributions

Conceived and designed the experiments: AA TM-D SO. Performed the experiments: ND RPC TM-D KD MT. Analyzed the data: TM-D IM. Contributed reagents/materials/analysis tools: AA TM-D. Wrote the paper: TM-D SO AA DD.

References

1. Aina O, Dadik J, Charurat M, Amangaman P, Gurumdi S, et al (2005) Reference values of CD4 T lymphocytes in human immunodeficiency virus-negative adult Nigerians. Clin Diagn Lab Immunol 12(4): 525–30. Available from: http://cvi.asm.org/cgi/content/abstract/12/4/525.
2. Oladepo DK, Idigbe EO, Audu Ra, Inyang US, Imade GE, et al (2009) Establishment of reference values of CD4 and CD8 lymphocyte subsets in healthy Nigerian adults. Clin Vaccine Immunol 16(9): 1374–7. Available from: http://www.pubmedcentral.nih.gov/articlerender.fcgi?artid = 2745013&tool = pmcentrez&rendertype = abstract.
3. Bain BJ (1996) Ethnic and sex differences in the total and differential white cell count and platelet count. J Clin Pathol 49(8): 664–6. Available from: http://www.pubmedcentral.nih.gov/articlerender.fcgi?artid = 500612&tool = pmcentrez&rendertype = abstract.
4. Saathoff E, Schneider P, Kleinfeldt V, Geis S, Haule D, et al (2008) Laboratory reference values for healthy adults from southern Tanzania. Trop Med Int Health 13(5): 612–25. Available from: http://www.ncbi.nlm.nih.gov/pubmed/18331386.
5. Kibaya RS, Bautista CT, Sawe FK, Shaffer DN, Sateren WB, et al (2008) Reference ranges for the clinical laboratory derived from a rural population in Kericho, Kenya. PLoS One 3(10): e3327. Available from: http://www.pubmedcentral.nih.gov/articlerender.fcgi?artid = 2553265&tool = pmcentrez&rendertype = abstract.
6. Lugada ES, Mermin J, Kaharuza F, Ulvestad E, Were W, et al (2004) Population-Based Hematologic and Immunologic Reference Values for a Healthy Ugandan Population 11(1): 29–34.
7. Tugume SB, Piwowar EM, Lutalo TOM, Mugyenyi PN, Grant RM, et al (1995) Hematological Reference Ranges among Healthy Ugandans 2(2): 233–5.
8. Mine M, Moyo S, Stevens P, Michael K, Novitsky V, et al (2011) Immunohaematological reference values for HIV-negative healthy adults in Botswana. Afr J Lab Med 1(1): 5–11. Available from: http://www.ajlmonline.org/index.php/ajlm/article/view/5.
9. Eller LA, Eller MA, Ouma B, Kataaha P, Kyabaggu D, et al (2008) Reference Intervals in Healthy Adult Ugandan Blood Donors and Their Impact on Conducting International Vaccine Trials 3(12).
10. Zeh C, Amornkul PN, Inzaule S, Ondoa P, Oyaro B, et al (2011) Population-based biochemistry, immunologic and hematological reference values for adolescents and young adults in a rural population in Western Kenya. PLoS One 6(6): e21040. Available from: http://www.pubmedcentral.nih.gov/articlerender.fcgi?artid = 3119664&tool = pmcentrez&rendertype = abstract.
11. Kratz A, Ferraro M (2004) Laboratory reference values. New Engl J 1548–63. Available from: https://qatar-weill.cornell.edu/elibrary/coursesupport/bod/NEJMLabReferenceValues.pdf.
12. Urassa WK, Mbena EM, Swai AB, Gaines H, Mhalu FS, et al (2003) Lymphocyte subset enumeration in HIV seronegative and HIV-1 seropositive adults in Dar es Salaam, Tanzania: determination of reference values in males and females and comparison of two flow cytometric methods. J Immunol Methods 277(1–2): 65–74. Available from: http://www.sciencedirect.com/science/article/pii/S0022175903001741.
13. Karita E, Ketter N, Price Ma, Kayitenkore K, Kaleebu P, et al (2009) CLSI-derived hematology and biochemistry reference intervals for healthy adults in eastern and southern Africa. PLoS One 4(2): e4401. Available from: http://www.pubmedcentral.nih.gov/articlerender.fcgi?artid = 2632744&tool = pmcentrez&rendertype = abstract.
14. Nkrumah K (2007) ORIGINAL ARTICLES POPULATION BASED REFERENCE INTERVALS FOR COMMON BLOOD HAEMATOLOGICAL AND BIOCHEMICAL PARAMETERS IN.; 41(4): 160–6.
15. Gill GV, England A, Marshal C (1979) Low platelet counts in Zambians. Transactions of the Royal Society of Tropical Medicine and Hygiene 73(1), 111–112.
16. Azikiwe AN (1984) Platelet count values in healthy Nigeria medical students in Jos. East African medical journal 61(6), 482–485.
17. Shaper AG, Lewis P (1971) GENETIC NEUTROPENIA IN PEOPLE OF AFRICAN ORIGIN. Lancet 298(7732): 1021–3. Available from: http://www.sciencedirect.com/science/article/pii/S0140673671903357.
18. Badenhorst CJ, Fourie J, Steyn K, Jooste PL, Lombard CJ, et al (1995) The haematological profile of urban black Africans aged 15–64 years in the Cape Peninsula. East African medical journal 72 (1), 19–24.
19. Dosoo DK, Kayan K, Adu-Gyasi D, Kwara E, Ocran J, et al (2012) Haematological and biochemical reference values for healthy adults in the middle belt of Ghana. PLoS One 7(4): e36308. Available from: http://www.pubmedcentral.nih.gov/articlerender.fcgi?artid = 3338654&tool = pmcentrez&rendertype = abstract.
20. Ngowi BJ, Mfinanga SG, Bruun JN, Morkve O (2009) Immunohaematological reference values in human immunodeficiency virus-negative adolescent and adults in rural northern Tanzania. BMC Infect Dis 9: 1. Available from: http://www.pubmedcentral.nih.gov/articlerender.fcgi?artid = 2630915&tool = pmcentrez&rendertype = abstract.
